# Author Correction: Genome evolution and the emergence of pathogenicity in avian *Escherichia coli*

**DOI:** 10.1038/s41467-021-22238-5

**Published:** 2021-03-22

**Authors:** Leonardos Mageiros, Guillaume Méric, Sion C. Bayliss, Johan Pensar, Ben Pascoe, Evangelos Mourkas, Jessica K. Calland, Koji Yahara, Susan Murray, Thomas S. Wilkinson, Lisa K. Williams, Matthew D. Hitchings, Jonathan Porter, Kirsty Kemmett, Edward J. Feil, Keith A. Jolley, Nicola J. Williams, Jukka Corander, Samuel K. Sheppard

**Affiliations:** 1 The Milner Centre for Evolution, Department of Biology and Biochemistry, University of Bath, Claverton Down, Bath, UK; 2 MRC Cloud Infrastructure for Microbial Bioinformatics (CLIMB) Consortium, London, UK; 3 Department of Biostatistics, University of Oslo, Oslo, Norway; 4 Department of Mathematics and Statistics, Helsinki Institute for Information Technology, University of Helsinki, Helsinki, Finland; 5 Antimicrobial Resistance Research Centre, National Institute of Infectious Diseases, Tokyo, Japan; 6 Uppsala University, Department for medical biochemistry and microbiology, Uppsala University, Uppsala, Sweden; 7 Swansea University Medical School, Institute of Life Science, Swansea, SA2 8PP UK; 8 National Laboratory Service, Environment Agency, Starcross, UK; 9 Department of Epidemiology and Population Health, Institute of Infection & Global Health, University of Liverpool, Leahurst Campus, Wirral, UK; 10 Department of Zoology, University of Oxford, South Parks Road, Oxford, OX1 3PS UK; 11 Parasites and Microbes, Wellcome Sanger Institute, Cambridge, UK

**Keywords:** Evolutionary genetics, Comparative genomics, Bacterial evolution, Bacterial pathogenesis

Correction to: *Nature Communications* 10.1038/s41467-021-20988-w, published online 3 February 2021.

The original version of this Article contained errors related to phylogroup attribution of lineages ST-117, ST-657, ST-1163, ST-4673, ST-23, ST-88 and ST-2230.

The ‘Results’ originally incorrectly read ‘Poultry isolates from our dataset (Supplementary Data 1) clustered within six out of the eight known *E. coli* phylogroups (A, B1, B2, D, E, F)^43^, and were absent in phylogroups C and G^44,45^’. The correct version replaces this sentence with ‘Poultry isolates from our dataset (Supplementary Data 1) clustered within the eight known *E. coli* phylogroups (A, B1, B2, C, D, E, F and G)^43-45^’.

The ‘Results’ omitted to mention that lineage ST-117 belongs to phylogroup G in the sentence: ‘In a second analysis, we tested the ability of the model trained on data from phylogroup A, B1 and B2 isolates, to predict infection status (healthy carriage vs. disease) in the emergent ST-117 lineage’. The correct version replaces this text with ‘In a second analysis, we tested the ability of the model trained on data from phylogroup A, B1 and B2 isolates, to predict infection status (healthy carriage vs. disease) in the emergent ST-117 lineage (phylogroup G)’.

The ‘Discussion’ originally incorrectly read ‘In fact, disease isolates were distributed across the phylogeny within all six previously described phylogroups and the ST-117 complex lineage’. The correct version replaces this sentence with ‘In fact, disease isolates were distributed across the phylogeny within all eight previously described phylogroups’.

The original versions of Figs. 1 and 3 omitted to mention that lineage ST-117 belongs to phylogroup G.

The original version of Fig. 3a omitted labels for phylogroups C and G and incorrectly labelled isolates from phylogroup C as belonging to phylogroup B1.

All the errors have been corrected in both the PDF and HTML versions of the Article.

In addition, the original version of the Supplementary Information associated with this Article included an incorrect Supplementary Data 1 file, in which phylogroup attribution of some lineages was omitted or incorrect. The HTML has been updated to include a corrected version of Supplementary Data 1, in which lineages ST-117, ST-657, ST-1163 and ST-4674 are correctly classified within phylogroup G and lineages ST-23, ST-88 and ST-2230 are correctly classified within phylogroup C. The correct version of Supplementary Data 1 can be found as Supplementary Information associated with this Correction.

## Supplementary information

Supplementary Data 1

