# Supplementary material for: Genome evolution and the emergence of pathogenicity in avian Escherichia coli
Source: Nat Commun. 2021 Feb 3;12:765. doi: 10.1038/s41467-021-20988-w (PMC7858641; doi:10.1038/s41467-021-20988-w)
Supplement: Supplementary file 1 — Supplementary Information [file 41467_2021_20988_MOESM1_ESM.pdf]

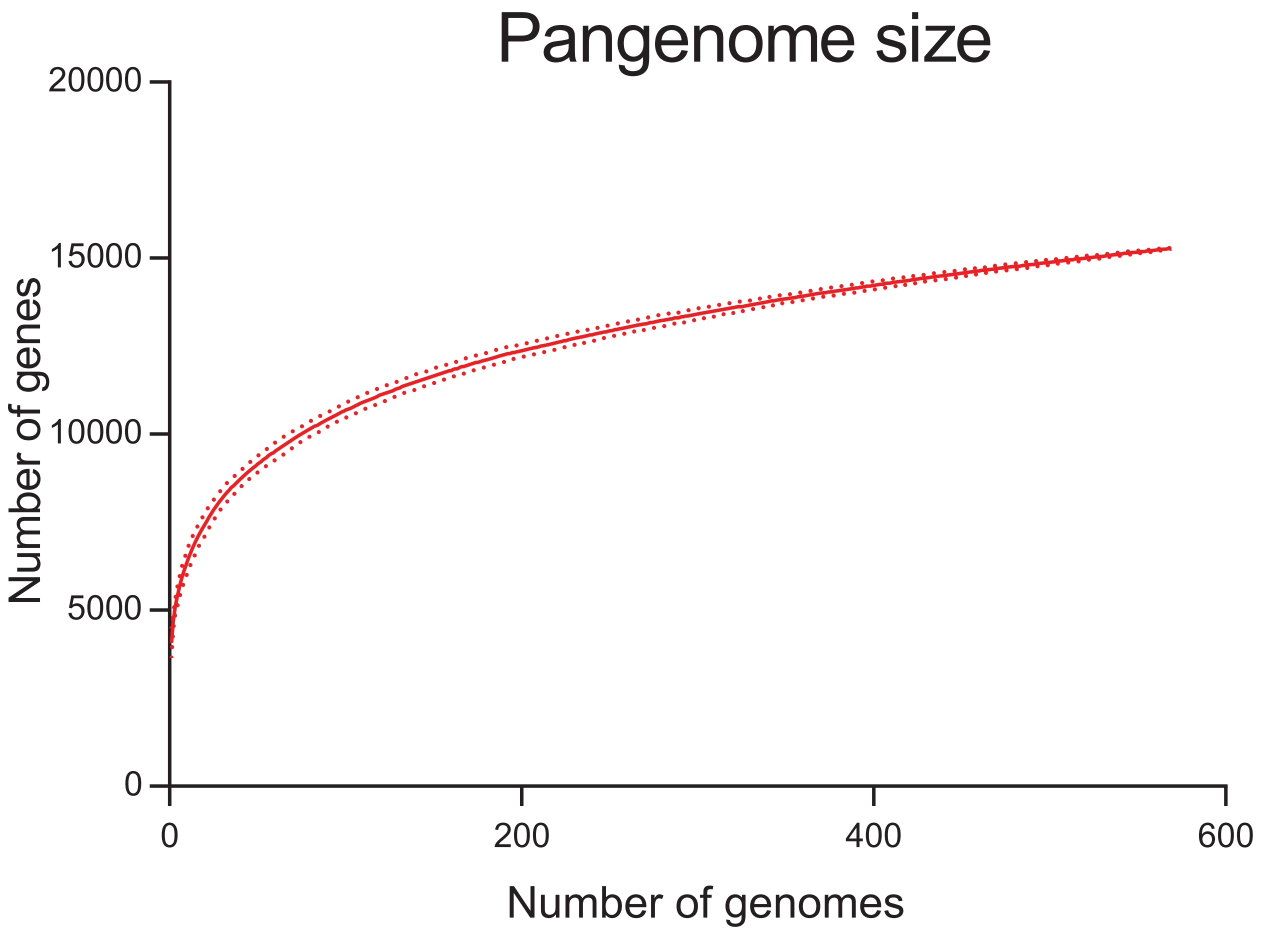

Supplementary Figure 1. Accumulation curve estimate of the avian *E. coli* pangenome

The total number of genes was determined as genome sampling increased. Comparisons were made based on a gene presence/absence matrix, derived from the pangenome list of the *E. coli* genomes in this study. Randomized genome sampling was carried out 100 times to obtain average number of genes for each sample comparison number (plain line) and standard deviation (dotted line).

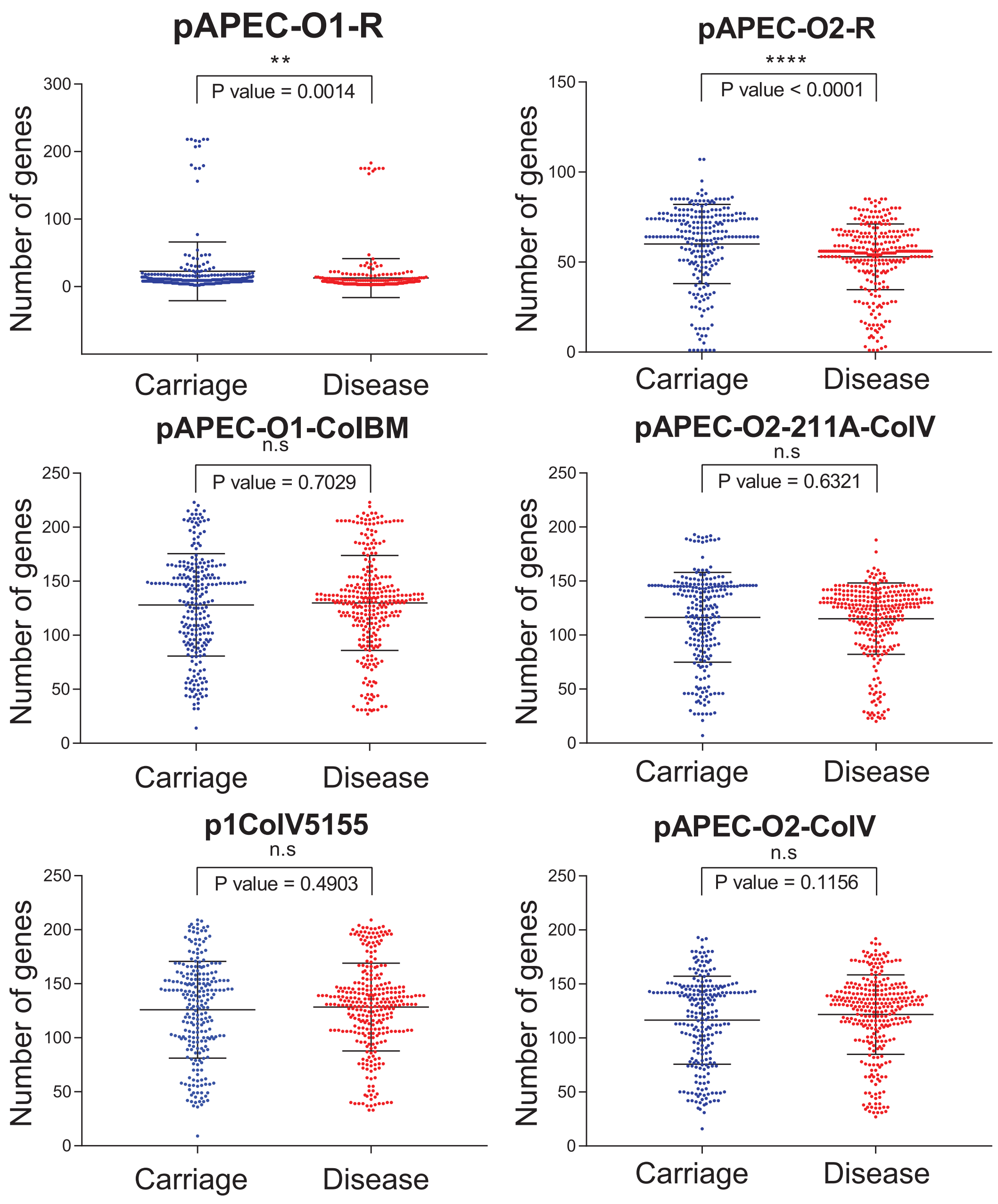

Supplementary Figure 2. Distribution of the genes of six known APEC plasmids between carriage and disease isolates

The frequency of known APEC-associated plasmid genes was calculated for carriage (blue, n = 234) and disease isolates (red, n = 307). For each of the six known APEC plasmids, the mean values +/- SD are shown with p-value determined using two-tailed t-test, n.s indicates not significant and significant differences are denoted by (*) $p < 0.05$, (**) $p < 0.01$, (***) $p < 0.001$, (****) $p < 0.0001$. Source data are provided as a Source Data file.

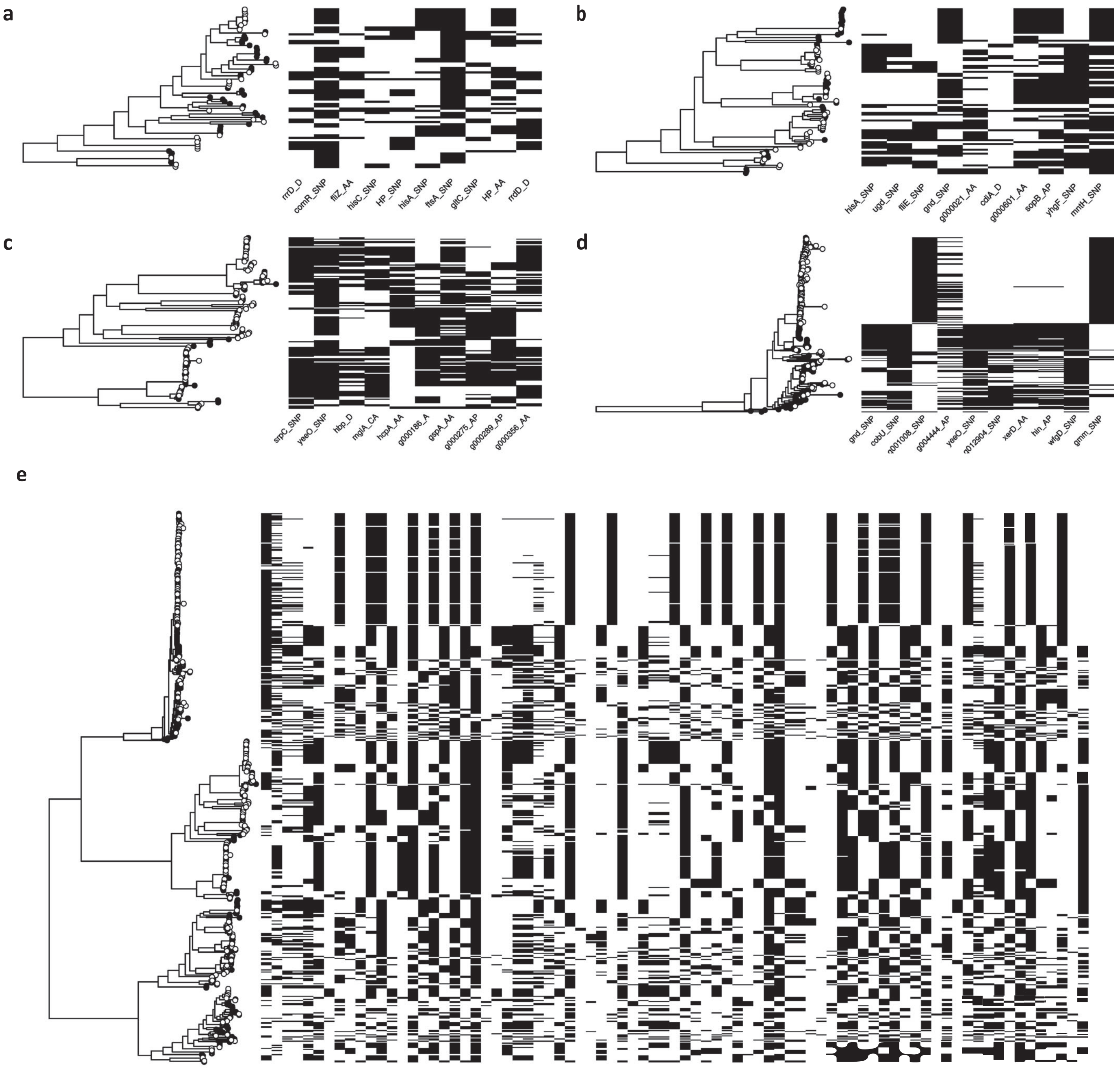

Supplementary Figure 3. Lineage-specific and species-wide disease-associated elements.

ClonalFrameML trees were constructed from recombination-masked alignments for: phylogroups A (a), B1 (b), B2 (c), the ST-117 clonal complex (d), and the dataset as a whole (e). In each case isolates from disease (white) and carriage (black) are indicated, as is the presence (black bars) of the 10 most significant disease-associated elements in the GWAS analysis corresponding to that dataset.

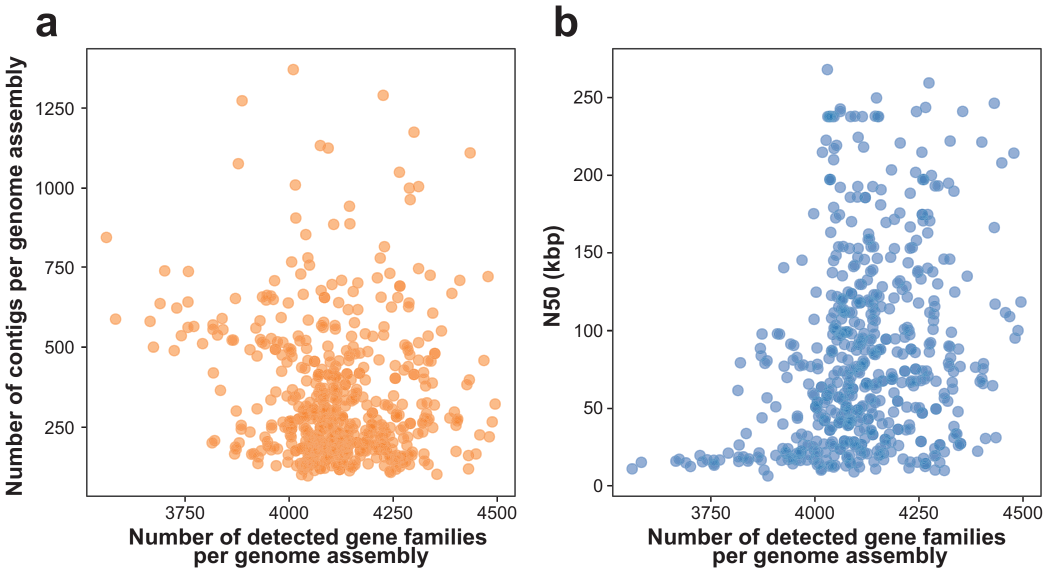

Supplementary Figure 4. Weak correlation between assembly quality metrics and the number of genes detected using the concatenated gene-by-gene alignment approach.

The number of detected gene families was compared against two assembly quality metrics: (a) total number of contigs per genome assembly and (b) N50 values, defined as the minimum contig length (in kbp) needed to cover 50% of the genome. Gene families were detected using the PIRATE pipeline. This visualization is based on values reported in Table S4, with complete genomes excluded (*n* = 3, strains APECO1, APECO78 and 53C.1). Source data are provided as a Source Data file.

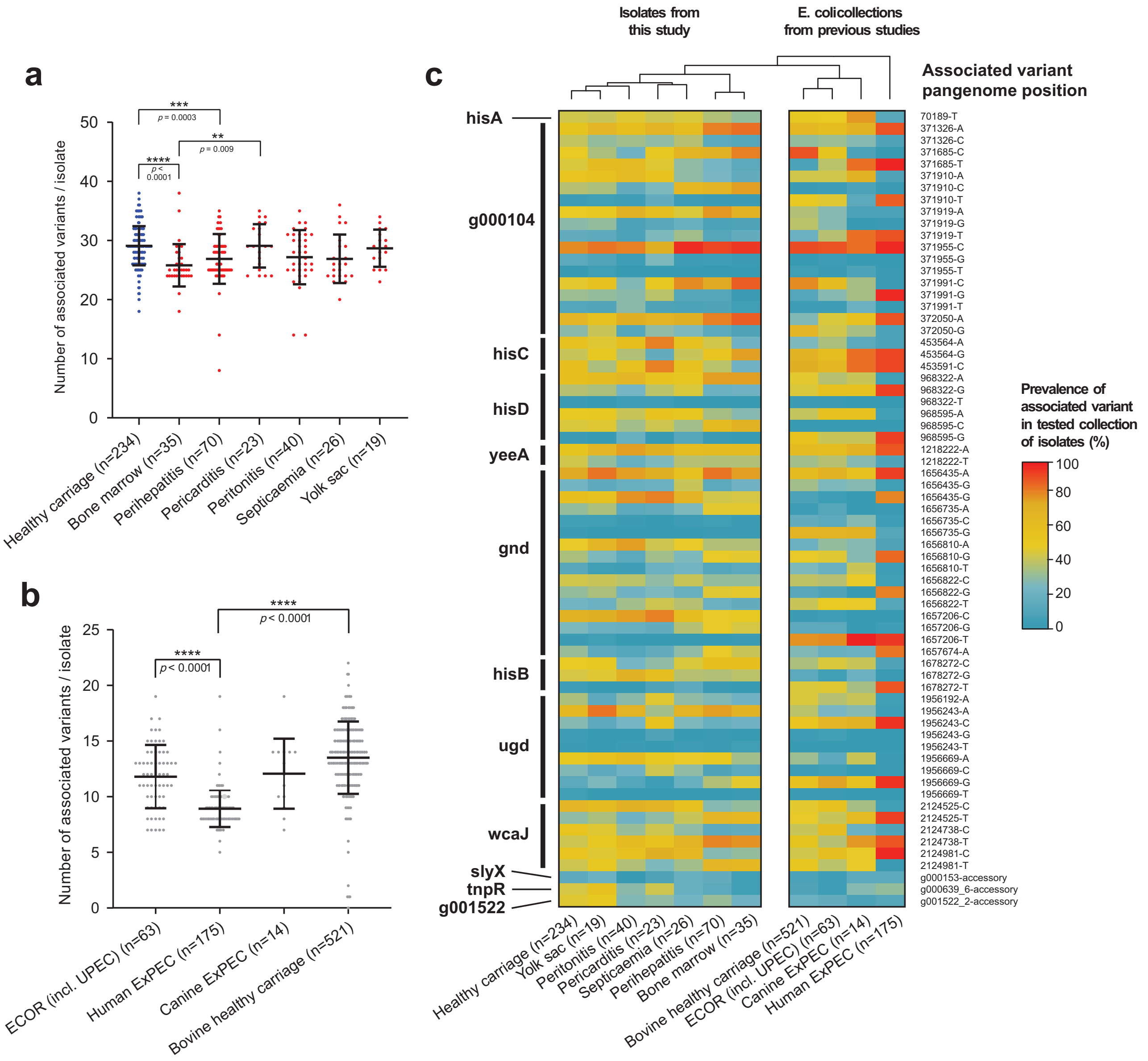

Supplementary Figure 5. Prevalence of species-wide disease-associated genetic variants in *E. coli* isolates from different infection sites and other host sources.

The prevalence of global APEC-associated genetic variants (common to 4 independent bacterial GWAS experiments) among (a) isolates from this study including 234 from asymptomatic carriage and 213 from 6 extraintestinal infection sites and (b) Isolates from previously published studies including 62 isolates from the E. coli reference collection (ECOR)[42], 175 human ExPEC[28], 14 canine ExPEC[51] and 521 healthy carriage isolates from cattle[52]. For each of the groups, the mean values +/- SD are shown with *p*-value determined using a Kruskal-Wallis test. Significant differences are indicated by (*) $p < 0.05$, (**) $p < 0.01$, (***) $p < 0.001$, (****) $p < 0.0001$. (c) Heatmap illustrating the prevalence of individual disease-associated SNPs among isolates from different infection sites and published collections. The dendrogram indicates hierarchical clustering of the profiles from compared isolate collections, performed using the complete linkage method. Source Data are provided as a Source Data file.
