# Supplementary material for: Genome evolution and the emergence of pathogenicity in avian Escherichia coli
Source: Nat Commun. 2021 Feb 3;12:765. doi: 10.1038/s41467-021-20988-w (PMC7858641; doi:10.1038/s41467-021-20988-w)
Supplement: Supplementary file 3 — Description of Additional Supplementary Files [file 41467_2021_20988_MOESM3_ESM.pdf]

Description of Additional Supplementary Files

File Name: Supplementary Data 1
Description: *E. coli* isolates used in this study

File Name: Supplementary Data 2
Description: List of 143 genes associated with infection in avian *E.coli*

File Name: Supplementary Data 3
Description: List of the 79 overlapping genomic variants used as pathogenicity predictors in the random forest models

File Name: Supplementary Data 4
Description: Assembly metrics for isolates used in this study

File Name: Supplementary Data 5
Description: Characterization of putative plasmid contigs using PlasmidFinder 2.1134

File Name: Supplementary Data 6
Description: Prevalence of APECassociated genetic variants used in the RF model within four different *E. coli* datasets

File Name: Supplementary Data 7
Description: Prevalence of APECassociated genetic variants used in the RF model in isolates from different infection sites
