# Supplementary material for: Genome evolution and the emergence of pathogenicity in avian Escherichia coli
Source: Nat Commun. 2021 Feb 3;12:765. doi: 10.1038/s41467-021-20988-w (PMC7858641; doi:10.1038/s41467-021-20988-w)
Supplement: Supplementary file 11 — Reporting Summary [file 41467_2021_20988_MOESM11_ESM.pdf]

nature research

Corresponding author(s): Samuel K Sheppard

Last updated by author(s): Oct 15, 2020

Reporting Summary

Nature Research wishes to improve the reproducibility of the work that we publish. This form provides structure for consistency and transparency in reporting. For further information on Nature Research policies, see our Editorial Policies and the Editorial Policy Checklist.

Statistics

For all statistical analyses, confirm that the following items are present in the figure legend, table legend, main text, or Methods section.

n/a	Confirmed	
☐	☒	The exact sample size (n) for each experimental group/condition, given as a discrete number and unit of measurement
☒	☐	A statement on whether measurements were taken from distinct samples or whether the same sample was measured repeatedly
☐	☒	The statistical test(s) used AND whether they are one- or two-sided<br>*Only common tests should be described solely by name; describe more complex techniques in the Methods section.*
☐	☒	A description of all covariates tested
☒	☐	A description of any assumptions or corrections, such as tests of normality and adjustment for multiple comparisons
☐	☒	A full description of the statistical parameters including central tendency (e.g. means) or other basic estimates (e.g. regression coefficient) AND variation (e.g. standard deviation) or associated estimates of uncertainty (e.g. confidence intervals)
☐	☒	For null hypothesis testing, the test statistic (e.g. F, t, r) with confidence intervals, effect sizes, degrees of freedom and P value noted<br>*Give P values as exact values whenever suitable.*
☒	☐	For Bayesian analysis, information on the choice of priors and Markov chain Monte Carlo settings
☒	☐	For hierarchical and complex designs, identification of the appropriate level for tests and full reporting of outcomes
☒	☐	Estimates of effect sizes (e.g. Cohen's d, Pearson's r), indicating how they were calculated

Our web collection on statistics for biologists contains articles on many of the points above.

Software and code

Policy information about availability of computer code

Data collection	Isolate genome and metadata was archived in BIGdb. The BIGSdb software is written in Perl, also utilizing some client-side Javascript. It runs on Linux using the Apache web server and PostgreSQL database. BIGSdb is open-source software, published under the GNU General Public Licence version 3
Data analysis	SPAdes (version 3.10.0) - de novo sequence assembly<br>PROKKA (version 1.13) and PIRATE (v1.0.4) - unique gene identification and automated annotation<br>SNIPPY (v4.6.0) - pseudoread simulation and mapping to reference genome.<br>IQtree (version 1.6.8) - Maximum-likelihood phylogenies construction<br>Microreact (https://microreact.org/showcase) - tree visualization<br>ClonalFrameML (v1.12) - Inference of putative recombination sites<br>cfml-maskrc (https://github.com/kwongj/cfml-maskrc) - masking recombination sites<br>treeWAS (https://github.com/caitiecollins/treeWAS) - genome wide association studies<br>Abricate 0.8.13 - plasmid gene annotation<br>R Phangorn (v2.5.5, R package) - consistency index calculation<br>RandomForest (v4.6-14), ROCR (v1.0-11), and ggplot2 (v3.3.2) (R packages) - Random forest analysis

For manuscripts utilizing custom algorithms or software that are central to the research but not yet described in published literature, software must be made available to editors and reviewers. We strongly encourage code deposition in a community repository (e.g. GitHub). See the Nature Research guidelines for submitting code & software for further information.

Data

Policy information about availability of data

All manuscripts must include a data availability statement. This statement should provide the following information, where applicable:

- Accession codes, unique identifiers, or web links for publicly available datasets
- A list of figures that have associated raw data
- A description of any restrictions on data availability

Short-read sequence data for all isolates sequenced in this study are deposited in the sequence read archive (SRA) and can be found associated with BioProject #PRNJA592536 (https://www.ncbi.nlm.nih.gov/bioproject/PRJNA592536). Assembled genomes are also available on Figshare (X DOI: 10.6084/m9.figshare.12011811). NCBI genome accession numbers for isolates in the validation dataset are included in Supplementary Data 1.

Field-specific reporting

Please select the one below that is the best fit for your research. If you are not sure, read the appropriate sections before making your selection.

☐ Life sciences ☐ Behavioural & social sciences ☒ Ecological, evolutionary & environmental sciences

For a reference copy of the document with all sections, see nature.com/documents/nr-reporting-summary-flat.pdf

Ecological, evolutionary & environmental sciences study design

All studies must disclose on these points even when the disclosure is negative.

Study description	Combining population scale comparative genomics and pangenome-wide association studies, we compared E. coli from commensal carriage and systemic infections. We identify phylogroup-specific and species-wide genetic elements that are enriched in APEC, including pathogenicity-associated variation in 143 genes that have diverse functions. Associated elements recorded in multiple strains achieved greater p-value for significance.
Research sample	The research sample included bacterial isolates sampled from poultry (symptomatic and asymptomatic) and the farm environment.
Sampling strategy	Sample size was determined through comparison with published bacterial GWAS studies by the authors and others (Meric et al, Nat Commun, 2018). The aim of the sampling effort was to collect enough samples in the majority of E. coli phylogroups (> 60) to be able to perform GWAS with statistically meaningful results in each clade.
Data collection	Isolates were collected from poultry and the farm environment by the authors through collaboration with an industrial partner. Isolate metadata was recorded by the authors. Isolate genomes were augmented with published genomes. Healthy associated strains were collected using fresh faecal swabs from asymptomatic poultry. Disease associated strains were collected from a postmortem examination of recently dead birds. This procedure was coordinated by Nicola J. Williams (Kristy Kemmett et al, PLOS ONE, 2013).
Timing and spatial scale	Isolates were sampled from 1982-2017 at the following frequencies: 1982, 2; 1983, 2; 1987, 1; 1990, 2; 2006, 2; 2008, 2; 2009, 5; 2010, 5; 2011, 1; 2012, 109; 2013, 87; 2014, 146; 2015, 144; 2016, 46; 2017, 14.<br>Isolates were sampled from different locations (mainly poultry farms) at the following frequencies: 44 from Denmark, 11 from Finland, 12 from France, 11 from Norway, 19 from Sweden, 389 from the United Kingdom, 73 from the United States and 9 from various other countries.<br>Summarizing, we consider our collection as a broad overview of asymptomatic and APEC isolates from multiple farms, and countries for over 35 years. Additionally our disease associated strains were isolated from various infection points displaying a range of APEC symptoms aiming to provide a genetic overview of the E. coli causing infection.
Data exclusions	No isolates were excluded.
Reproducibility	We performed four independent GWAS analysis in four distinct E. coli phylogroups. This study focuses on the 143 genes that were flagged as pathogenicity associated in all four GWAS analyses (Supplementary Data 2). The results of each GWAS can be replicated by re-running the treeWAS algorithm in the same phylogroups using the isolates of each phylogroup.
Randomization	Throughout the study, isolate organization into groups was principally carried out based upon metadata. Specifically isolation source: disease associated strains from postmortem examination of recently deceased birds, and healthy carriage strains from faecal isolates of asymptomatic poultry. Randomization and covariate analysis are not applicable in GWAS because phenotype (source) data is necessarily included a priori in order to detect elements that are over represented in the groups under investigation.
Blinding	This is relevant to association studies but it is not possible in GWAS experiments because phenotype (source) data is necessarily included a priori in order to detect elements that are over represented in one group or another (compared to expected frequencies based upon the tree).

Did the study involve field work? ☐ Yes ☒ No

Reporting for specific materials, systems and methods

We require information from authors about some types of materials, experimental systems and methods used in many studies. Here, indicate whether each material, system or method listed is relevant to your study. If you are not sure if a list item applies to your research, read the appropriate section before selecting a response.

Materials & experimental systems

n/a	Involved in the study
☒	☐ Antibodies
☒	☐ Eukaryotic cell lines
☒	☐ Palaeontology and archaeology
☐	☒ Animals and other organisms
☒	☐ Human research participants
☒	☐ Clinical data
☒	☐ Dual use research of concern

Methods

n/a	Involved in the study
☒	☐ ChIP-seq
☒	☐ Flow cytometry
☒	☐ MRI-based neuroimaging

Animals and other organisms

Policy information about studies involving animals; ARRIVE guidelines recommended for reporting animal research

Laboratory animals	Not involved
Wild animals	Bacteria were sampled from chickens that were routinely slaughtered for human consumption.
Field-collected samples	Samples were not taken from live animals in the field.
Ethics oversight	University of Bath.

Note that full information on the approval of the study protocol must also be provided in the manuscript.
